# Influence of Low Sintering Temperature on BaCe_0.2_Zr_0.6_Y_0.2_O_3−δ_ IT-SOFC Perovskite Electrolyte Synthesized by Co-Precipitation Method

**DOI:** 10.3390/ma15103585

**Published:** 2022-05-17

**Authors:** Muhammad Rafique, Neelam Safdar, Muneeb Irshad, Muhammad Usman, Maaz Akhtar, Muhammad Wajid Saleem, Muhammad Mujtaba Abbas, Ahmed Ashour, Manzoore Elahi Soudagar

**Affiliations:** 1 Department of Physics, University of Sahiwal, Sahiwal 57000, Pakistan; 2 Department of Physics, University of Gujrat, Gujrat 50700, Pakistan; neelamsafdar800@gmail.com; 3 Department of Physics, University of Engineering and Technology, Lahore 54890, Pakistan; muneebirshad@gmail.com; 4 Department of Mechanical Engineering, University of Engineering and Technology, Lahore 54890, Pakistan; muhammadusman@uet.edu.pk; 5 Mechanical Engineering Department, NED University of Engineering and Technology, Karachi 75270, Pakistan; maaz@neduet.edu.pk; 6 Department of Mechanical and Mechatronics Engineering, College of Engineering, Dhofar University, Salalah 211, Oman; msaleem@du.edu.om; 7 Department of Mechanical Engineering, New Campus, University of Engineering and Technology, Lahore 39021, Pakistan; 8 Engineering Mathematics and Physics Department, Faculty of Engineering and Technology, Future University in Egypt, New Cairo 11845, Egypt; ahmed.ashour@fue.edu.eg; 9 Department of Mechanical Engineering and University Centre for Research & Development, Chandigarh University, Mohali 140413, Punjab, India; me.soudagar@gmail.com

**Keywords:** BCZY, electrolyte, perovskite, co-precipitation

## Abstract

BaCe_0.2_Zr_0.6_Y_0.2_O_3−δ_ (BCZY) perovskite electrolytes were synthesized for intermediate-temperature solid oxide fuel cell with a cost-effective and versatile co-precipitation method. The synthesized BCZY electrolytes were sintered at 900, 1000, and 1100 °C to observe the effects of low sintering temperature on the structural, morphological, thermal, and electrical properties of BCZY. All BCZY electrolytes materials exhibited a crystalline perovskite structure and were found to be thermally stable. The crystallinity and conductivity of BCZY electrolyte enhanced with increased sintering temperature, due to the grain growth. At the same time, secondary phases of carbonates were also observed for samples sintered at a temperature lower than 1100 °C. The BCZY sintered at 1100 °C exhibited a density >95%, and a power density of 350 mWcm^−2^ with open-circuit voltage 1.02 V at 650 °C was observed due its dense and airtight structure. Based on the current investigation, we suggest that the BaCe_0.2_Zr_0.6_Y_0.2_O_3−δ_ perovskite electrolyte sintered at a temperature of 1100 °C is a suitable electrolyte for IT-SOFC.

## 1. Introduction

Fuel cells have gained a lot of interest during the last decade as an alternative energy resource. Much focus is devoted to making them efficient and cost-effective [1,2]. Solid oxide fuel cells have received much consideration, among others, because of their fuel flexibility, inexpensive catalyst, and efficiency [3,4]. SOFCs have a solid electrolyte between two electrodes: cathode and anode [5,6,7]. SOFCs operating at high temperatures (at about 1000 °C) give high conductivities (>10^−1^ Scm^−1^) and efficiencies, thus limiting their commercial usage [3,8,9,10]. Therefore, reducing the operating temperature will help with the stability, performance, efficiency, and commercialization of SOFCs [11]. Reducing operating temperature assists in terms of manufacturing costs, material stability, and operational cost, but resistive losses across the solid electrolyte and overpotential at electrodes cause an increase in operating temperatures. Three approaches are commonly used to reduce these losses: (i) decrease in electrolyte thickness, (ii) usage of materials having high conductivities at low operating temperatures, and (iii) usage of electrodes with low polarization resistance [12,13,14].

The electrolyte is a key component of SOFCs that greatly affects their performance; therefore, developments in electrolyte materials are crucial in lowering the working temperature of SOFCs with better chemical stability and high ionic conductivity [15,16,17,18]. Many ionic conductors, such as ZrO_2_, LaGaO_3_, YSZ, and CeO_2_, have been investigated as electrolytes in SOFCs. Still, these electrolytes require a high activation energy and temperature to provide maximum conductivity [19]. On the other hand, proton conductors are promising candidates for SOFC electrolytes since they have high protonic conductivity at low temperatures [20,21]. BCZYs from cerate and zirconate families are considered promising perovskite electrolytes because, at low temperatures, they exhibit high proton conductivity [20,22,23]. Ni et al. observed that barium cerate (BaCeO_3_) electrolytes exhibit high ionic conductivity that can be enhanced to 10^−2^ Ω^−1^cm^−1^ by (15–20%) doping of yttrium. However, their chemical stability suffered due to the presence of hydrocarbons at the anode site that react with carbon dioxide, resulting in the poor performance of the cell [24]. Barium zirconate (BaZrO_3_), on the other hand, shows greater stability in a CO_2_ atmosphere but exhibits low ionic conductivity [25]. Therefore, composite electrolytes of BaCeO_3_ and BaZrO_3_ were developed, exhibiting both high conductivity and chemical stability. It is also reported that BCZY electrolytes-based SOFCs had shown high short-term power densities of about 0.4 and 1.0 Wcm^−2^ at 600 and 700 °C [26,27,28]. Yuqing Meng et al. synthesized BaCe_0.7_Zr_0.1_Y_0.2−x_Sm_x_O_3−δ_ electrolyte materials by using the ball-milling process and sintering at 1400 °C. The reported performance was 410 mWcm^−2^ [29]. Another group of researchers also fabricated a BCZY electrolyte with dopants (2 mol% Fe, Ni) and sintered it at 1400 °C. The electrochemical performance obtained was 450 mWcm^−2^ [30]. Kang et al. prepared BaCe_0.7_Zr_0.2_Y_0.2_ (3 mol% Ni as sintering aid) and sintered it at 1200 °C. The performance obtained was 106 mWcm^−2^ [31]. The use of a sintering aid also gained considerable interest for the lowering of the sintering temperature. Recently, Zaheer ud Din et al. sintered BaCe_0.7_Zr_0.1_Y_0.2_O_3−δ_ (BCZY) perovskite electrolytes at 1150 °C, using sintering aids (CuO-Bi_2_O_3_), and improved the electrochemical performance of a proton-conducting electrolyte that was observed at a previously reported high temperature [32].

The synthesis routes are crucial in attaining the required properties of the material. The same material synthesized with different methods will exhibit a different structure and morphology. The optimization of the sintering temperature for each route is therefore necessary to obtain a material of desired properties and densification. The Co-precipitation synthesis route, among other wet chemical routes, is considered to be a versatile, simple, and cost-effective route to obtain fine and uniformly shaped powder material [12,13,14,32].

In the current project, the lowest possible sintering temperature was optimized for a BCZY (BaCe_0.2_Zr_0.6_Y_0.2_O_3−δ_) electrolyte synthesized via a cost-effective co-precipitation method. The upshots of a varying sintering temperature on structural, morphological, electrical, and thermal properties were investigated through different characterizations.

## 2. Experimental Details

BaCe_0.2_Zr_0.6_Y_0.2_O_3−δ_ powder electrolyte was synthesized by using Na_2_CO_3_ as a precipitating agent through one-step co-precipitation. Y(NO_3_)_3_.6H_2_O (>99%), Ba(NO_3_)_2_ (>99%), Ce(NO_3_)_3_·6H_2_O (>99%), and Zr(NO_3_)_4_·5H_2_O (>99%) were used as precursor materials. The nitrate salts were dissolved in deionized water under continuous stirring and heating. A separate sodium carbonate solution (Na_2_CO_3_) was formed. The molar ratio of sodium carbonate and metal cations was adjusted to 1:1 to precipitate the metal cations. The carbonate solution was added dropwise in nitrate solution, with continuous stirring at 90 °C, and the filtration process collected white precipitates, which were dried at 150 °C for 2 h. The dried powder was then sintered in a vacuum furnace at 900, 1000, and 1100 °C temperatures for 4 h, with at a rate of 3 °C/min. The sintered powder was then grinded and pressed into pellets for analysis.

An X-ray diffractometer analyzed the structural phases and crystallinity of BCZY electrolyte powder. The surface morphology was investigated through SEM (JEOL, JSM 6360). The thermal stability up to 900 °C in air was observed by SETARAM, with a rate of 10 °C/min. The density was measured by using the Archimedean immersion method. The sides of the pellets were coated with silver paste for measuring ionic conductivity, using the four-point probe DC method.

## 3. Results

### 3.1. XRD Analysis

**Figure 1:**
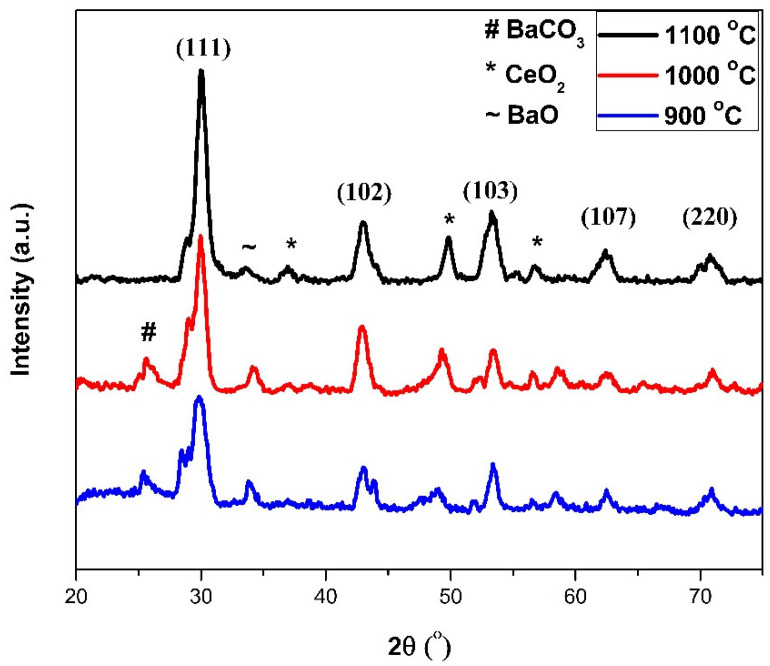
XRD spectra of sintered BaCe_0.2_Zr_0.6_Y_0.2_O_3−δ_ electrolyte.

Figure 1 shows the XRD patterns of the synthesized BaCe_0.2_Zr_0.6_Y_0.2_O_3−δ_ electrolytes sintered at 900, 1000, and 1100 °C. The XRD patterns represent the diffraction peaks at 30°, 42°, 53°, 63°, and 71°, corresponding to (111), (102), (103), (107), and (220) planes, respectively. These planes indicate the cubic phase of BCZY the composite (JCPDS Nos. 89-2486 and 82-2372).

It is clear from the spectra that, with an increased sintering temperature, the shape of the peaks (e.g., reflection (111)) sharpened, depicting increased crystallinity of the samples. The crystallinity increased due to improvement in grain growth with temperature. An increase in the sintering temperature enhanced grain boundary movement, resulting in an increased crystallite size [33].

It is also clear from the spectra that secondary phases of barium carbonate (BaCO_3_), ceria (CeO_2_), and barium oxide exist in BCZY electrolyte material at a low sintering temperature and decreased with increased temperature. This is because of the decomposition of barium carbonate and barium oxide, which start decomposing above 1000 °C in temperature. It is also reported that it can be decomposed completely above a temperature of 1100 °C [34]. Moreover, it is reported that pure BCZY phases can be achieved at high sintering temperatures, due to the decomposition and evaporation of secondary phases; therefore, a high sintering temperature is recommended to attain a proper phase with increased crystallinity.

The crystallite size of the sintered samples was calculated by using Scherrer’s equation [35]:

(1) $$L=\frac{K\lambda }{\beta \cos\theta }$$

The calculated average crystallites size was 10.48, 12.681, and 18.51 nm for 900, 1000, and 1100 °C, respectively. The increased crystallite size is because of the fact that boundaries of grain migrated more quickly with the increased sintering temperature.

### 3.2. Density Measurements

The density of the sintered BaCe_0.2_Zr_0.6_Y_0.2_O_3−δ_ electrolytes was measured by using the XRD parameters and Archimedes method. The BCZY has a theoretical density of 6.18 gcm^−3^. The experimental density was calculated by using the Archimedes principle, using equation [36]:

(2) $$D=W_{1}\;⍴/W_{1}-W_{2}$$

where ⍴ is the density of water used as fluid (water at 25 °C is 0.997 gcm^−3^), *W*_1_ is the weight of the sample in air, and *W*_2_ is the wet weight (in water) [37]. The average relative density was 83%, 90%, and 95% for electrolytes sintered at 900, 1000, and 1100 °C, respectively. The sample sintered at 900, 1000, and 1100 °C showed 17%, 10%, and 5% porosity, respectively. Hence, the sample sintered at 1100 °C exhibited a dense structure, limiting the leakage of the ions and, thus, making it an efficient electrolyte.

### 3.3. Surface Morphology

**Figure 2:**
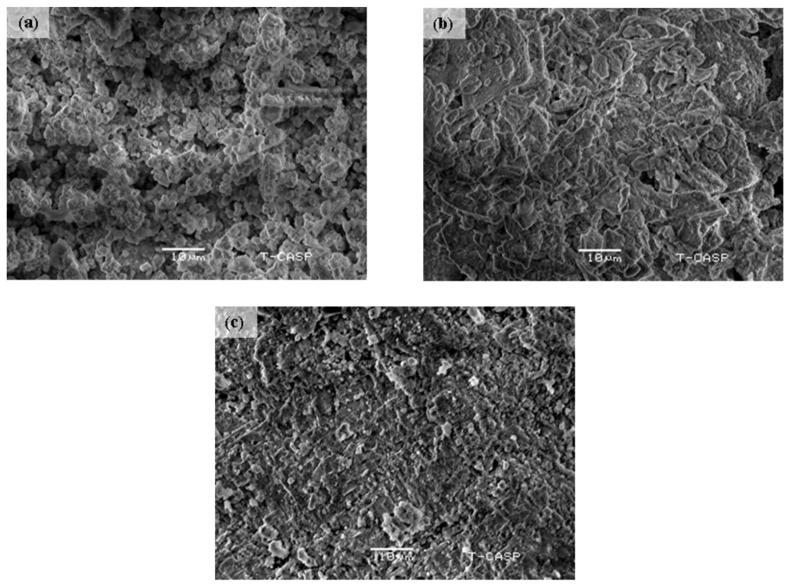
Micrographs of sintered BaCe_0.2_Zr_0.6_Y_0.2_O_3_-_δ_ electrolyte: (**a**) 900 °C, (**b**) 1000 °C, and (**c**) 1100 °C.

Figure 2 shows the micrographs of sintered BaCe_0.2_Zr_0.6_Y_0.2_O_3−δ_ electrolytes. The SEM micrographs clearly show that the surface morphology of BCZY changed with an increased sintering temperature. It can also be observed that pores disappeared with the increased temperature because of diffusion kinetics (mass transport at higher sintering for increase of further bond area and mechanical strength) and recrystallization, which resulted in increased grain size and decreased grain boundaries [38]. Furthermore, the porosity reduced gradually with the rise in temperature, as confirmed by the Archimedes’ principle, too. Large-sized grains with dense and compact structures can be observed for materials sintered at 1100 °C in Figure 2c, fulfilling the main requirement of SOFC electrolyte: the electrolyte should be free of pores to prevent diffusion of gases from one side to the other side of the electrolyte.

### 3.4. Thermal Stability Analysis

**Figure 3:**
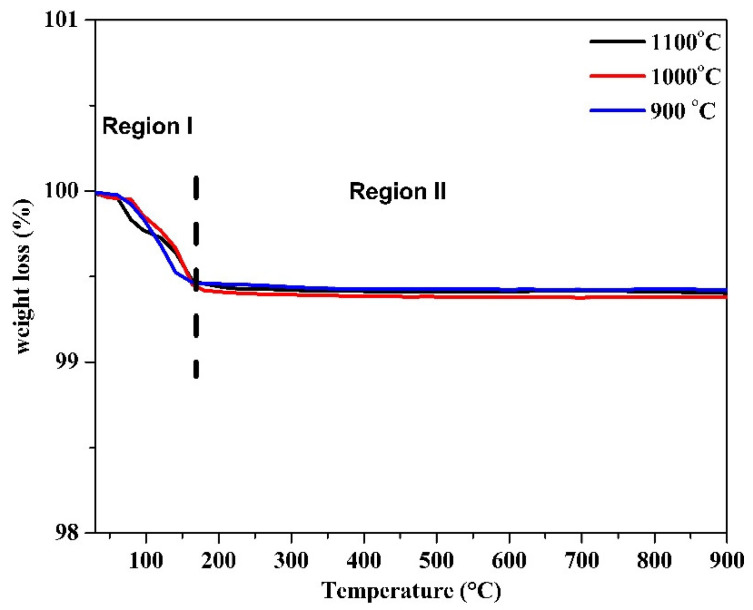
TGA curves for sintered BaCe_0.2_Zr_0.6_Y_0.2_O_3−δ_ electrolyte.

The TGA curves in Figure 3 give the temperature-dependent weight loss of sintered BaCe_0.2_Zr_0.6_Y_0.2_O_3−δ_ electrolytes. It is clear from the plot that a very small weight loss occurred because the analysis was performed after the sintering, which already evaporated and decomposed the water, nitrate, or organic compounds present within the synthesized materials. Similar behavior was also reported by a group of researchers earlier [39]. The TGA curve in each sample can be categorized into two regions: (i) 30 to 160 °C and (ii) 160 to 900 °C. In Region I (30 to 160 °C), a very small loss occurred due to the evaporation and decomposition of absorbed water in the BaCe_0.2_Zr_0.6_Y_0.2_O_3−δ_ electrolytes from the moisture of the ambient environment [40]. In Region II (160 to 900 °C), there was no weight loss observed under heating, thus suggesting that no volatilization or decomposition reactions occurred between 160 and 900 °C; therefore, it will remain chemically stable at the SOFC operating temperature.

### 3.5. Conductivity

**Figure 4:**
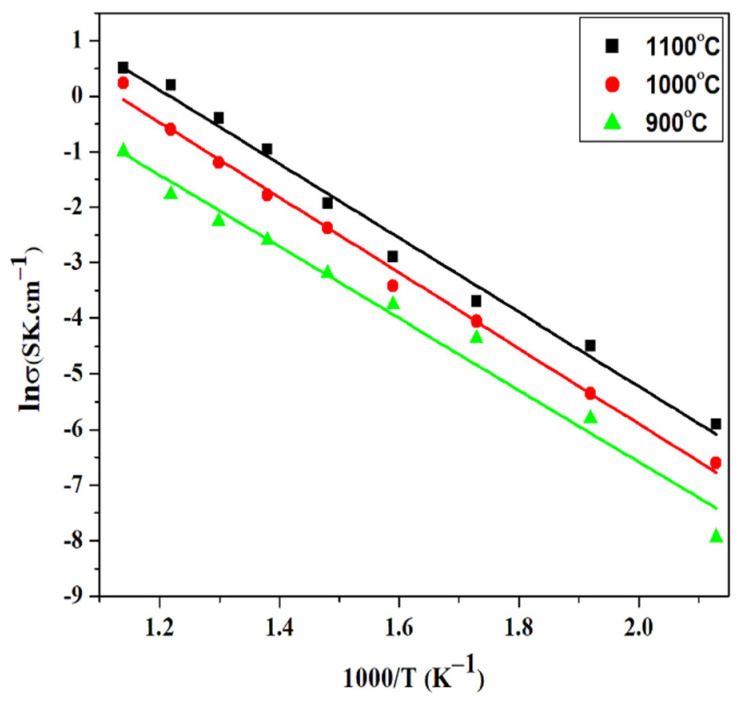
Conductivity curves of sintered BaCe_0.2_Zr_0.6_Y_0.2_O_3−δ_ electrolyte.

The measured ionic conductivity of the synthesized BCZY electrolytes is shown in the Arrhenius plot in Figure 4. It was calculated by using the Arrhenius relation [39]:

(3) $$\alpha =\frac{\alpha_{{}^{\circ}}}{T}\exp\frac{E_{a}}{KT}\;$$

Figure 4 depicts that conductivity increased as the sintering rose from 900 to 1100 °C. This increased conductivity is because of improved grain growth and microstructural changes. The densification and reduction of pores enhanced the rate of the ions’ diffusion through the grain boundaries. Moreover, the secondary phases were minimized due to the increase in temperature, which reduced the electrolyte loses and gas inter-diffusion, resulting in increased conductivity [41]. The activation energy of 0.53 eV was obtained for the electrolyte sintered at 1100 °C. Emiliana et al. reported an activation energy of 0.66–0.68 eV for Y and Pr doped barium zirconate sintered at 1500 °C [42]. Nur syafkeena et al. reported an activation energy of 0.79 eV for the BaCe_0.54_Zr_0.36_Y_0.1_O_3−δ_ electrolyte sintered at 1500 °C [43]. However, low activation energy (0.41 eV) for BCZY (BaCe_0.7_Zr_0.1_Y_0.2_O_3−δ_) sintered at 1150 °C was also reported when CuO-Bi_2_O_3_ (2 mol%) was used as a sintering aid [32].

### 3.6. Cell Performance

**Figure 5:**
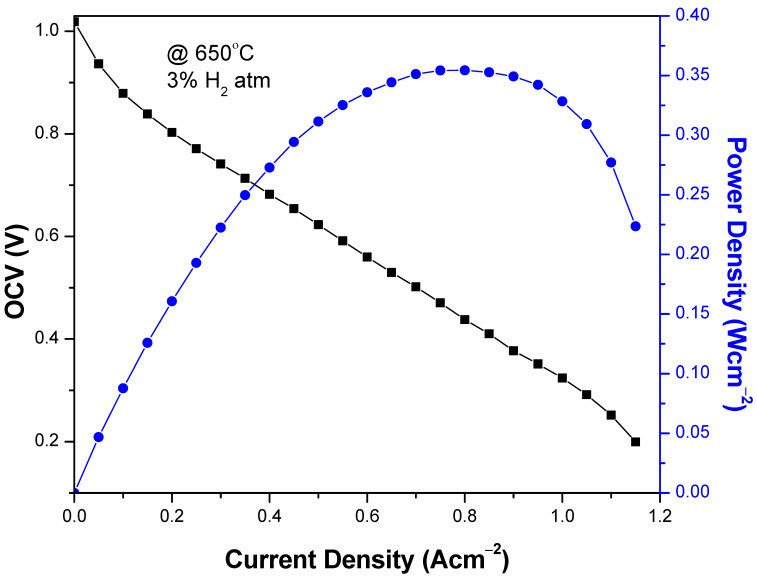
Electrochemical performance of cell with BaCe_0.2_Zr_0.6_Y_0.2_O_3_-_δ_ electrolyte sintered at 1100 °C.

The electrochemical performance of a single cell consisting of Ni-BCZY/BCZY (sintered at 1100 °C)/BCZY-LSCF was evaluated in a 3% H_2_ atmosphere at 650 °C and is shown in Figure 5. The BaCe_0.2_Zr_0.6_Y_0.2_O_3−δ_ perovskite electrolyte material sintered at 1100 °C was used as an electrolyte, due to its high density and conductivity value. Moreover, it was observed in XRD that the barium carbonate phase decomposed at this temperature; therefore, it is more suited for the electrochemical evaluations among all samples. The maximum power density obtained was 350 mW/cm^2^, with an OCV of 1.02 V. The measured performance of the BCZY electrolyte sintered at 1100 °C is comparable to the electrolytes sintered at high temperatures [44,45].

The performance can be enhanced by increasing the sintering temperature up to 1300 °C, which is still lower compared to the reported temperatures. The secondary phases will be removed by the high sintering temperature, and, therefore, single-phase BCZY will give a better electrochemical performance. A comparative table for the performance of the SOFC with the BCZY electrolyte is given in Table 1.

## 4. Discussion

The structural analysis (Figure 1) confirmed the cubic phase of BaCe_0.2_Zr_0.6_Y_0.2_O_3−δ_ (BCZY) perovskite electrolytes (JCPDS Nos. 89-2486 and 82-2372). The structural analysis also showed increased crystallinity of the samples with increasing sintering temperature, because of enhanced grain growth at high temperatures. The calculated average crystallites size was 10.48, 12.681, and 18.51 nm for BaCe_0.2_Zr_0.6_Y_0.2_O_3−δ_ perovskite electrolytes sintered at 900, 1000, and 1100 °C, respectively. The increase in sintering temperature caused better grain boundary movement because grain boundaries migrate more quickly with increased temperature, resulting in increased crystallite size. Secondary planes of barium carbonate (BaCO_3_), ceria (CeO_2_), and barium oxide (BaO) are also observed in BaCe_0.2_Zr_0.6_Y_0.2_O_3−δ_ electrolytes. The intensity of these secondary planes decreased with increased temperature. The decomposition of barium carbonate and barium oxide starts above 1000 °C temperature, and it is also reported that it decomposes completely at a temperature >1100 °C.

The density of the synthesized BaCe_0.2_Zr_0.6_Y_0.2_O_3−δ_ perovskite electrolytes was calculated by the Archimedean principle. The BaCe_0.2_Zr_0.6_Y_0.2_O_3−δ_ electrolytes sintered at 900, 1000, and 1100 °C showed 17%, 10%, and 5% porosity, respectively, depicting that BaCe_0.2_Zr_0.6_Y_0.2_O_3−δ_ sintered at 1100 °C exhibited a dense structure, thus limiting the leakage of the ions and making it an efficient electrolyte.

The surface morphology of BaCe_0.2_Zr_0.6_Y_0.2_O_3−δ_ perovskite electrolytes revealed that pores disappeared with the increased temperature because of diffusion kinetics and recrystallization. Furthermore, the porosity also reduced gradually with the temperature rise, as confirmed by Archimedes’ principle. Large-sized grains with dense and compact structures can be observed for material sintered at 1100 °C in Figure 2c, fulfilling the main requirement of SOFC electrolyte: the electrolyte should be free of pores to prevent diffusion of gases.

Our thermal analysis (Figure 3) showed a small weight loss because it was performed after the sintering, which already evaporated and decomposed the water, nitrate, or organic compounds present in BaCe_0.2_Zr_0.6_Y_0.2_O_3−δ_ perovskite electrolytes. The weight loss occurred only from 30 to 160 °C because of evaporation and decomposition of absorbed water present in BaCe_0.2_Zr_0.6_Y_0.2_O_3−δ_. The presence of a small amount of water was attributed to the moisture of the ambient environment. It can be inferred that sintered BaCe_0.2_Zr_0.6_Y_0.2_O_3−δ_ electrolytes remained chemically stable from room temperature to 900 °C.

Figure 4 depicts that, with an increased sintering temperature (900 to 1100 °C), the conductivity of BaCe_0.2_Zr_0.6_Y_0.2_O_3−δ_ perovskite electrolytes increased. This increased conductivity is because of improved grain growth and microstructural changes. The densification and reduction of pores enhanced the rate of ions diffusion through grain boundaries. Moreover, secondary phases were minimized due to the increase in temperature, which reduced the electrolyte losses and gas inter-diffusion, resulting in increased conductivity. The highest conductivity was observed for the BaCe_0.2_Zr_0.6_Y_0.2_O_3−δ_ electrolyte sintered at 1100 °C, having an activation energy of 0.53 eV. Figure 5 shows the electrochemical performance of the button cell comprising Ni-BCZY/BCZY (sintered at 1100 °C)/BCZY-LSCF. The BaCe_0.2_Zr_0.6_Y_0.2_O_3−δ_ electrolyte sintered at 1100 °C is used as an electrolyte due to its high density and conductivity value. The power density obtained was 350 mW/cm^2^ with OCV of 1.02V at 650 °C, suggesting that the BaCe_0.2_Zr_0.6_Y_0.2_O_3−δ_ electrolyte sintered at 1100 °C can be utilized as SOFC. However, a high performance can be yielded if the sintering temperature of the BaCe_0.2_Zr_0.6_Y_0.2_O_3−δ_ composition synthesized by co-precipitation is increased up to 1300 °C.

## 5. Conclusions

The perovskite BCZY (BaCe_0.2_Zr_0.6_Y_0.2_O_3−δ_) electrolyte powder was successfully synthesized by using the co-precipitation method. The temperature was kept at 90 °C during the synthesis, and the synthesis was completed in under two hours. The prepared electrolyte was then sintered at 900, 1000, and 1100 °C to enhance the structural, morphological, electrical, and thermal properties. BCZY exhibited the crystallite structure, and the crystallite size increased from 10.48 to 18.51 nm with the increased sintering temperature. Secondary phases (e.g., BaCO_3_) were minimized with increased temperature because of their decomposition at high temperatures; therefore, higher than 1100 °C is required for better performance. SEM micrographs depicted that the porosity decreased, and the structure became denser by raising the temperature from 900 to 1100 °C, because of increased diffusion kinetics, resulting in increased grain size. Furthermore, BCZY exhibited thermal stability as an electrolyte from 160 to 900 °C. The conductivity of BCZY increased with the sintering temperature because of grain growth and densification, which avoided the gas cross-diffusion. A power density of 350 mW/cm^2^ was obtained for the cell that had a BCZY electrolyte sintered at 1100 °C. It can be deduced that the composition BaCe_0.2_Zr_0.6_Y_0.2_O_3−δ_ sintered at a temperature of 1100 °C is a potential candidate for IT-SOFC electrolyte material; however, sintering the current composition at a temperature greater than 1100 °C for 4–5 h can significantly enhance the overall cell performance.

## Figures and Tables

**Table 1:** Power Densities of SOFC with different compositions of BCZY electrolyte [46].

Electrolyte	BaCe_0.4_Zr_0.4_Y_0.2_O_3−δ_	BaCe_0.5_Zr_0.35_Y_0.15_O_3−δ_	BaZr_0.4_Ce_0.4_Y_0.2_O_3−δ_	BaZr_0.1_Ce0_0.7_Y_0.2_O_3−δ_
Cathode	PrNi	LSC	BSCF	LSCF
Anode	Ni-BCZY	Ni-BCZY	Ni-BCZY	Ni-BCZY
Temperature (°C)	550	550	600	600
Power Density (mW cm^−2^)	63	300	360	477

## Data Availability

Not applicable.
